# Senolytics in idiopathic pulmonary fibrosis: Results from a first-in-human, open-label, pilot study

**DOI:** 10.1016/j.ebiom.2018.12.052

**Published:** 2019-01-05

**Authors:** Jamie N. Justice, Anoop M. Nambiar, Tamar Tchkonia, Nathan K. LeBrasseur, Rodolfo Pascual, Shahrukh K. Hashmi, Larissa Prata, Michal M. Masternak, Stephen B. Kritchevsky, Nicolas Musi, James L. Kirkland

**Affiliations:** a Sticht Center for Healthy Aging and Alzheimer's Prevention, Internal Medicine – Gerontology and Geriatric Medicine, Wake Forest School of Medicine (WFSM), 1 Medical Center Blvd, Winston-Salem, NC 27157, United States; b Division of Pulmonary Diseases and Critical Care Medicine, Department of Internal Medicine, University of Texas Health Sciences Center at San Antonio (UTHSCSA) and South Texas Veterans Health Care System, San Antonio, TX 78229, United States; c Robert and Arlene Kogod Center on Aging, Mayo Clinic, Rochester, MN 55905, United States; d Internal Medicine – Pulmonary, Critical Care, Allergy, Immunologic Medicine, Wake Forest School of Medicine, 1 Medical Center Blvd, Winston-Salem, NC 27157, United States; e Burnett School of Biomedical Sciences, University of Central Florida, Orlando, FL 32827, United States; f Barshop Institute for Longevity and Aging Studies, Center for Healthy Aging, University of Texas Health Sciences Center at San Antonio and South Texas Veterans Health Care System, San Antonio, TX 78229, United States; g San Antonio Geriatric Research, Education and Clinical Center, South Texas Veterans Health Care System, San Antonio, TX 78229, United States

**Keywords:** Cellular senescence, Senolytics, Translation, Clinical trial, Interstitial lung disease, Idiopathic pulmonary fibrosis, Aging

## Abstract

**Background:**

Cellular senescence is a key mechanism that drives age-related diseases, but has yet to be targeted therapeutically in humans. Idiopathic pulmonary fibrosis (IPF) is a progressive, fatal cellular senescence-associated disease. Selectively ablating senescent cells using dasatinib plus quercetin (DQ) alleviates IPF-related dysfunction in bleomycin-administered mice.

**Methods:**

A two-center, open-label study of intermittent DQ (D:100 mg/day, Q:1250 mg/day, three-days/week over three-weeks) was conducted in participants with IPF (*n* = 14) to evaluate feasibility of implementing a senolytic intervention. The primary endpoints were retention rates and completion rates for planned clinical assessments. Secondary endpoints were safety and change in functional and reported health measures. Associations with the senescence-associated secretory phenotype (SASP) were explored.

**Findings:**

Fourteen patients with stable IPF were recruited. The retention rate was 100% with no DQ discontinuation; planned clinical assessments were complete in 13/14 participants. One serious adverse event was reported. Non-serious events were primarily mild-moderate, with respiratory symptoms (*n* = 16 total events), skin irritation/bruising (n = 14), and gastrointestinal discomfort (*n* = 12) being most frequent. Physical function evaluated as 6-min walk distance, 4-m gait speed, and chair-stands time was significantly and clinically-meaningfully improved (*p* < .05). Pulmonary function, clinical chemistries, frailty index (FI-LAB), and reported health were unchanged. DQ effects on circulat.ing SASP factors were inconclusive, but correlations were observed between change in function and change in SASP-related matrix-remodeling proteins, microRNAs, and pro-inflammatory cytokines (23/48 markers *r* ≥ 0.50).

**Interpretation:**

Our first-in-humans open-label pilot supports study feasibility and provides initial evidence that senolytics may alleviate physical dysfunction in IPF, warranting evaluation of DQ in larger randomized controlled trials for senescence-related diseases.

ClinicalTrials.gov identifier: NCT02874989 (posted 2016–2018).

**Research in context**

**Evidence before this study**

Targeting fundamental mechanisms of aging has the potential to reduce the severity of multiple age-related diseases. Cellular senescence is a cell fate in which proliferating or differentiated non-dividing cells undergo replicative arrest and develop a pro-fibrotic, pro-inflammatory, and apoptosis-inducing senescence-associated secretory phenotype (SASP). Senescent cells acquire resistance to apoptosis and their own SASP. Senescence is a mechanism that has been shown to drive multiple age-related diseases, including idiopathic pulmonary fibrosis (IPF). The occurrence of IPF, a chronic, fibrotic lung disease, rises dramatically with advancing age. It is devastating, with a relentlessly progressive course, a median survival of <4 years, and limited treatment options. Aging processes have been implicated in IPF pathogenesis, with accumulation of senescent cells in the lung, fibrosis, matrix remodeling, inflammation, DNA damage, telomere attrition, and alveolar epithelial oxidative stress. Extent of expression of markers of cellular senescence in lungs of patients with IPF is associated with disease severity. Senolytic agents are drugs developed using a hypothesis-driven approach that selectively induce senescent cell apoptosis by transiently disabling the senescent cell anti-apoptotic pathways (SCAPs) that defend senescent cells against their own pro-apoptotic environment. The first senolytics discovered were Dasatinib (D) and Quercetin (Q), which, in combination (DQ), cause apoptosis of senescent vs. non-senescent cells in human tissue. DQ alleviates a range of age- and chronic disease-related conditions in mice, including physical dysfunction in progeroid and naturally-aged mice, vascular stiffness in high fat-fed and aged mice, hepatic steatosis, age-related osteoporosis, neurodegenerative disease modelling Alzheimer disease, and premature development of age-related diseases and early death caused by transplanting small numbers of senescent cells into younger mice. Senolytics extend remaining lifespan in old mice. Importantly with respect to human IPF, DQ reduces lung senescent cell burden and restores function in mice with pulmonary fibrosis induced by bleomycin inhalation. Intermittent treatment of IPF and other age-related conditions in mice with DQ is effective despite the short elimination half-life of D and Q (<6 h), likely because senescent cells do not divide and take weeks to over a month to re-accumulate. Senolytics do not need to be present continuously to occupy a receptor or affect an enzyme.

**Added value of the study**

We report the first clinical trial using DQ or other senolytics in humans. Clinical trial data using senolytics in the context of senescence-associated diseases such as IPF do not exist. Apart from lung transplantation, the extent of alleviation of dysfunction by anti-fibrotics and other interventions for IPF has been disappointing. Here, in a first-in-human, proof-of-concept open-label clinical trial, we tested the feasibility and potential impact of intermittently treating patients with stable mild to severe IPF with 9 oral doses of DQ over 3 weeks. Physical function and clinical parameters were measured before and 5 days after the last DQ dose, well beyond these drugs' elimination half-lives. Fourteen participants were enrolled, completed intermittent drug self-administration, and 100% completed the study. Statistically significant (within-subject) and clinically meaningful improvements in physical function following DQ treatment were seen despite the small number of participants and short study duration. Such improved physical function has not been reported in subjects in the placebo arms of other IPF trials. Although adverse events occurred, they were acceptable and primarily consistent with the underlying IPF diagnosis, study procedures, or known-off target effects of the study drugs (e.g., gastrointestinal discomfort, headache). No changes in laboratory tests suggestive of hepatic or renal toxicity were found, and pulmonary function was unchanged. While effects of DQ on circulating SASP factors were inconclusive in this initial study, correlations were seen between change in function and change in SASP factors.

**Implications of all the available evidence**

In this first-in-human trial of senolytics, our data indicate that short-term, intermittent administration of DQ may alleviate the physical dysfunction that accompanies IPF, as is the case with DQ treatment for pulmonary fibrosis induced by bleomycin in mice. These findings suggest that evaluation of DQ and other senolytics in larger randomized, controlled trials for IPF and other cellular senescence-associated conditions is feasible.

Alt-text: Unlabelled Box

## 1 Introduction

Occurrence of idiopathic pulmonary fibrosis (IPF), a chronic, progressive fibrotic lung disease, rises dramatically with advancing age [[1], [2], [3]]. It is a devastating disease with median survival of 3.8 years in newly-diagnosed adults over 60 years of age [4]. Aging is implicated in IPF pathogenesis, including accelerated aging of the alveolar epithelium, denoted by oxidative stress, telomere attrition, DNA damage, inflammation, and matrix remodeling [1,5]. Converging evidence suggests that the aging hallmark, cellular senescence, may be a central mechanism contributing to IPF [6,7]. Cellular senescence is a non-proliferative cell state that can entail a senescence-associated secretory phenotype (SASP) comprising cytokines, chemokines, pro-fibrotic factors, matrix metalloproteases (MMPs), factors causing stem/progenitor cell dysfunction, and growth factors that impose detrimental effects on the local and systemic environment [8,9]. Senescent cells and the SASP may fuel an increasingly pro-inflammatory fibrotic response driving lung deterioration and severe functional decline in IPF. Senescence biomarkers accumulate in the lungs of IPF patients, and higher expression of key senescence biomarkers, such as p16^INK4A^ (CDKN2A), DNA damage foci (γH2A.X), telomere dysfunction, and SASP factors are associated with increased disease severity [5,10].

Senolytic agents, drugs that selectively induce senescent cell apoptosis, were first developed using a hypothesis-driven approach [11,12]. The idea for developing agents to target senescent cells was based on a key study showing that age-related senescent cell burden in mice was reduced by both caloric restriction and a lifespan-extending mutation [13]. These delays in senescent cell accumulation occurred in tandem with increased healthspan, suggesting that pharmacologically targeting senescent cells might delay onset of senescence-related diseases such as IPF. Our hypothesis-driven drug discovery approach was based on the observations that senescent cells release pro-apoptotic factors as part of their SASP [[14], [15], [16]], yet resist apoptosis [17], leading us to test if these cells rely on pro-survival senescent cell anti-apoptotic pathways (SCAPs) to defend against their SASP and pro-apoptotic intracellular *milieu*. Several such SCAPs were identified from bioinformatics analyses of senescent vs. non-senescent human cells [8,11,18,19]. Senescent cells originating from different cell types depend for survival on different SCAPs. We identified which components of these SCAPs are essential for survival of human senescent vs. non-senescent cells in RNA interference studies. Next, we selected agents know to target these SCAP components and found they selectively induce apoptosis in human senescent but not non-senescent cultured cells.

Using our hypothesis-driven drug discovery approach, the first senolytics identified were Dasatinib (D) and Quercetin (Q), which are effective in combination (DQ) when administered to mice [11]. D is a tyrosine kinase inhibitor in clinical use for treating leukemias and Q is a natural product that targets BCL-2, insulin/IGF-1, and HIF-1α SCAP network components. The DQ combination is effective in eliminating cultured senescent cells originating from several different types of human and mouse cells [5,8,11,18,[20], [21], [22], [23], [24], [25]].

DQ was recently demonstrated to decrease senescent cell burden and the SASP in human tissues within 48 h by selectively causing apoptosis in senescent vs. non-senescent cells in freshly isolated explants [24]. DQ alleviates a range of age- and senescence-related disorders in mice, including age-related osteoporosis, hepatic steatosis, neurodegeneration in Tau^+^ mouse models of Alzheimer disease, age- and high fat-diet-induced vascular calcification and hyporeactivity, and radiation-induced muscle dysfunction [5,8,11,18,[20], [21], [22], [23], [24], [25]]. In older mice, senolytics delay age-related diseases as a group and substantially increase late life survival [24,26]. Moreover, senolytics prevent the physical dysfunction, accelerated onset of age-related diseases, and early death caused by transplanting small numbers of senescent cells into young mice [24,26]. Importantly with respect to IPF, DQ alleviates bleomycin-induced pulmonary fibrosis, causing improved pulmonary function, body composition, and physical function [5]. DQ also decreases the impaired exercise endurance due to aging and progerias in mice [11,24].

Collectively, these data highlight the therapeutic potential of senolytic therapies for multiple aging-related diseases and geriatric syndromes, including IPF. Herein, we performed a first-in-human, small-scale pilot clinical trial with the objective to assess the feasibility, acceptability, best methods, and measurement characteristics of potential study outcomes for the senolytic drug combination DQ in older participants with stable IPF. The primary endpoints were retention rates and completion rates for planned clinical assessments. Secondary endpoints were: 1) initial safety estimates and adverse event reports and 2) change in functional and reported health measures. Functional endpoints were the change from baseline in: 1) 6-min walk distance (6MWD), 4-m usual gait speed, timed 5-repitition chair-stands, short physical performance battery (SPPB), and grip strength; 2) pulmonary function: forced vital capacity (FVC); forced expiratory volume in 1-s (FEV1); 3) clinical lab-based frailty index (FI-LAB); and 4) patient reported quality of life, respiratory health, and fatigue. Exploratory associations of change in function with changes in circulating senescence-associated secretory phenotype (SASP) factors were evaluated.

## 2 Materials and methods

### 2.1 Overview of methods

Intermittent D (100 mg/day) plus Q (1250 mg/day) were orally administered over three consecutive days in three consecutive weeks (i.e., 9 total participant administered dosing days) at two outpatient clinical research centers using a single-arm, open-label design. We measured clinical chemistries before and after DQ and performed rigorous symptom questionnaires weekly for health-related quality of life and off-target effects to obtain preliminary evidence of safety and tolerability. We evaluated potential assessment tools probing function and biological activity of DQ: 1) physical function: 6MWD, 4-m gait speed, timed chair-stands, SPPB, and grip strength; 2) pulmonary function: FVC; FEV1; 3) FI-LAB. We explored associations of change in clinical and functional assessments with biological assays of a set of circulating SASP components assayed in plasma using multiplex discovery platforms. SASP factors that had been associated with frailty (IL6, MCP-1, activin A, apelin) [[27], [28], [29], [30]] or had been reported to have prognostic or diagnostic potential in IPF (MMP1, MMP7, osteopontin) [31,32] were further validated by single target enzyme-linked immunosorbent assays (ELISA). In addition, non-coding micro (mi)-RNAs released locally and systemically may be associated with inflammation and senescence [33]. For example, miRNAs miR-146a-5p and miR-34a-5p have recently has been acknowledged as part of SASP in a mouse model of lung fibrosis and long-lived dwarf mice [34,35]. Moreover, miRNA expression suppresses cytokine secretion and may restrain excessive SASP activity in primary human fibroblasts and HUVEC cultures [36,37], and could be modulated by intervention in humans [38].

### 2.2 Sample size estimates

Traditional sample size determination is made to ensure specific power to detect targeted treatment effects. However, the primary purpose of this pilot study was to evaluate study feasibility rather than drug efficacy [39,40]. Moreover, no clinical data exist on effects of senolytics in humans or patients with IPF: prior data are not available to derive efficacy-based sample size estimates. Therefore, pilot trial sample size estimation (*n* = 14) was based on medium standardized effect sizes (0.5) for a main trial designed with 90% power and two-sided 5% significance [41].

#### 2.2.1 Participants

The study was conducted at the Clinical Research Units (CRU) of Wake Forest School of Medicine (WFSM, *n* = 2) and the University of Texas Health Science Center San Antonio (UTHSCSA, *n* = 12), part of the South Texas Veterans Health Care System. A total of 17 patients aged ≥50 years with stable IPF were primarily recruited from a study physician's clinical practice (n = 12) or from the community by direct mailings, a hospital-supported website, or ClinicalTrials.gov (*n* = 3, WFSM). After comprehensive in-clinic screening, 3 candidates were excluded from the study and the remaining 14 were enrolled. The minority-ethnicity status of the participants was 12 non-Hispanic white and 2 Hispanic white. All study procedures complied with the Declaration of Helsinki and the informed consent and study documents were approved by the Institutional Review Boards of WFSM and UTHSCSA and were reviewed by Data and Safety Monitoring Boards (DSMB) at both clinical sites. The nature, benefits, and risks of the study were explained to the patient volunteers and their written informed consent was obtained prior to participation.

The enrolled participants all had confirmed diagnoses of IPF according to published consensus guidelines^4^ based on historical high-resolution computed tomography (HRCT) and/or surgical lung biopsy showing usual interstitial pneumonia (UIP), on stable (3 months) therapy with nintedanib (Ofev) or pirfenidone (Esbriet) or no therapy, had BMI within the range 19–35 kg/m^2^, were cognitively intact as indicated by a Montreal Cognitive Assessment score > 21, women were postmenopausal (12 months), and were willing to maintain their current activity level and remain geographically close to clinical site for the duration of the study period. Enrolled participants did not experience more than two moderate/severe IPF exacerbations resulting in new prescriptions for antibiotics/oral steroids or events associated with hospitalization or emergency room visits within the past year. Eligible participants were free of lung transplant, pulmonary hypertension or cor pulmonale confirmed by echocardiography or heart catheterization, myocardial infarction, angina, hospitalization for cardiac etiology, stroke or transient ischemic attack in the past 6 months, chronic heart failure, current or chronic history of liver disease, neurologic condition, drug or alcohol abuse in previous 5 years, QTc prolongation, low CBC, Glomerular Filtration Rate (GFR) <30 (mL/min/1.73 m^2^), or ALT >2xULN and bilirubin >1.5xULN. Participants were not taking anti-arrhythmic medications known to cause QTc prolongation or Coumadin or other anti-platelet or anti-coagulant medication. Participants had no current use of quinolone antibiotics or drugs metabolized by the same liver enzymes as D or Q. Patients recruited from the UTHSCSA site perform regular 6 min walk distance (6MWD) tests as part of their clinical care and all patients had completed 6MWD test within the previous 12 months, though differences in the research and clinical protocols prevent direct comparison of distance walked.

#### 2.2.2 Dosing

Following screening and baseline measures, all participants were administered DQ orally (D: 100 mg/day, Sprycel, Bristol Myers Squibb and Q: 250 mg capsules × 5/day quercetin phytosome Thorne Research *Sophora japonica* concentrate [leaf]/phosphatidylcholine complex from Sunflower). The dose of D selected is based on the FDA-approved dose for chronic administration to patients as effective for inducing apoptosis in human cancer cells. Q is a natural product. DQ was administered in conjunction with stable standard of care (nintedanib, pirfenidone, or none). Nintedanib and dasatinib are both tyrosine kinase inhibitors, though only dasatanib demonstrates senolytic action [11]. To avoid potential drug-drug interaction, nintedanib was withheld on dosing days with dasatinib, but was resumed on non-dosing days; pirfenidone was maintained. Senolytics were anticipated to be effective when given intermittently. Our intermittent dosing regimen was based on our studies in mice with pulmonary fibrosis [5], effects of DQ on different lung cell types in culture,^88,42^ senescent cell clearance rate from freshly isolated human tissue explants treated with DQ [24], peak concentrations and elimination half-lives of DQ in humans [43,44], and dose escalation studies in old Rhesus monkeys.

DQ was delivered on an intermittent schedule as 3 doses on 3 consecutive days followed by 4-day no-drug periods, repeated over 3 weeks, resulting in 9 total dosing days over the 3 week intervention period (Fig. 2). The initial dose was administered onsite. Adherence was checked and adverse event reports and symptom questionnaires were administered weekly by telephone. Reminder calls were made before each weekly course of DQ began. Verbal checks for adherence, symptoms, and event reports were conducted approximately 24 h following each weekly course of DQ self-administration (fourth day of each intervention week). The symptom questionnaires combined common symptoms identified for chemotherapeutic drugs (Chemotherapy Symptom Assessment Scale; C-SAS) and drugs targeting IPF. Nonserious adverse events were collected from this weekly symptom questionnaire, reviewed by the study physician, and reported as mild, moderate, or severe to according to the Medical Dictionary for Regulatory Activities (MedDRA) 16.1. Our event collection, classification, and reporting are consistent with Phase III clinical trials in IPF [45,46]. Following 3 weeks of administration, baseline study procedures were repeated. Follow-up testing was performed one and two weeks after drug course completion, which is well beyond the D and Q elimination half-lives, which are both <6 h [43,44]. All participants completed the study (100% retention).

#### 2.2.3 Assessments and biospecimen collection

All assessments and clinical chemistries were performed at WFSM or UTHSCSA CRU. Blood draws for clinical laboratories and biological measures were obtained following 12-h overnight fasts. Measures of physical and pulmonary function and questionnaires were obtained after ingesting a small snack. All pre-post biospecimen pairs collected at WFSM and UTHSCSA were shipped to Mayo Clinic for batch senescence-related assays.

### 2.3 Eligibility determination, safety labs, and biospecimen collection

Electrical activity of the heart and QTc prolongation were evaluated by ECG. Body height and weight and blood pressure were measured at each visit. Safety laboratory tests included complete blood count (CBC), lipid panel, HbA1c, complete metabolic panel, and hsCRP. A clinical laboratory-based frailty index (FI-LAB) was constructed as number of laboratories outside of reference range per the total number of laboratory measures [47]. SASP and inflammatory markers were not included in the FI-LAB index. Fasted serum and EDTA plasma (10 mL each) specimens were drawn and stored in 0.5–1 mL aliquots for batched analysis of biological measures of senescence and SASP factors (below).

### 2.4 Physical function assessments

Six Minute Walk Distance (6MWD): The 6MWD test is a well-established outcome that is valid and reproducible in patients with a wide range of physical function, predicts clinical events, and responds to interventions [48,49]. The 6MWD was conducted in an unobstructed hallway utilizing a standardized script. Total distance covered and number and duration of rests and symptoms were recorded. 4 m Gait Speed: Walking speed was assessed by asking the participants to walk at their usual pace over a 4 m course, with the faster of two walks used to compute walking speed. Chair-Stands: To evaluate time to complete 5-repition chair-stands, participants were asked to stand up and sit down on a straight-backed chair five times, as quickly as possible without using their arms. Time to complete was recorded. Short Physical Performance Battery (SPPB) Score: performance on 4 m gait speed and chair-stands tests (described above) and a balance test were scored and combined to derive the summary SPPB Score (0–12 with 12 being the best performance) [50]. For the test of standing balance, participants were asked to maintain balance in three positions, characterized by a progressive narrowing of base support. The SPPB score was based on the percent of maximum achieved in each domain, with higher scores indicative of better performance. Grip Strength: Handgrip strength was measured twice in each hand to the nearest 2 kg using an isometric Hydraulic Hand Dynamometer (Jamar, Bolingbrook, IL).

### 2.5 Pulmonary function assessment

Ventilatory capacity was assessed by spirometry recorded by an EasyOne™ PLUS spirometer to record average FEV1 over three reproducible trials. The essential elements of the test emphasized to the participant included: filling lungs completely, sealing lips around the spirette so there were no leaks, taking care not to block its opening with teeth or tongue or bite down excessively, breathing out as forcefully and rapidly as possible, and to continue blowing out until the lungs are completely empty. FEV1 is reported as liters per second (L/s) and as percent predicted value based on age, sex, race, and height. FVC is reported as liters (L) and as percent predicted value based on age, sex, race, and height. Ratio of percent predicted FEV1 to percent predicted FVC was calculated.

### 2.6 Patient-reported health measures

IPF-related symptoms were assessed by questionnaires, including the Modified Medical Research Council (mMRC), the Saint George Respiratory Questionnaire (SGRQ; respiratory health related quality of life), Patient's Global Impressions of Change (PGIC), and University of California San Diego Shortness of Breath Questionnaire (UCSD-SoBQ). Fatigue was assessed by the Pittsburgh Fatigability Scale (PFS) and the Fatigue Severity Scale (FSS).

#### 2.7 Biological measures of senescence and SASP factors

All assays to determine cellular senescence burden and SASP factors were performed under masked conditions using serum or EDTA-treated plasma collected before and after 3 weeks of DQ at UTHSCSA and WFSM. Post-DQ blood collections were conducted 5–7 days after the final DQ dose, well after the ~1/2 day half-lives of D and Q [43,44], and under fasted conditions. The proportion of senescent cells based on axillary skin biopsy was envisioned as a measure during trial planning (see clinicaltrials.govNCT02874989); however, technical complications resulted in omission from the present study.

### 2.7.1 Plasma and serum analyses

Plasma cytokines were quantified using multiplex ELISA on a Bio-Plex 200 analyzer by Eve Technologies (Calgary, Alberta, Canada) using Human Cytokine 42-plex, MMP9 plex, and TIMP 4-plex discovery assays. All samples were run in duplicates. Single targeted ELISA's (vendors below) were run to measure plasma osteopontin, apelin12, PAI-1, activin A, IL-6, and MCP-1, and serum MMP-1 and MMP-7 (Human Activin A: DAC008, R&D systems; Human Total MMP-7 [serum]: DMP700, R&D systems; Human IL-6: D6050, R&D systems; Human Serpin E1/PAI-1: DSE100, R&D systems; Human CCL2/MCP-1: DCP00, R&D systems; Human Osteopontin: BMS2066, eBioscience; Human Apelin-12: EK-057-23, Phoenix Pharmaceuticals; Human MMP-1 (serum): ELH-MMP-1, RayBiotech., Inc.).

### 2.7.2 MicroRNA analyses

Total miRNAs were isolated from serum using the miRNEasy Serum/Plasma kit (Qiagen, Cat No. 217184) adjusting the manufacturer's instructions to allow for 400 μL of sample volume. All samples were spiked-in with 3.5 μL of control Ce_miR39_1, provided by the manufacturer (Lot no. 237024736). cDNA was synthesized using the TaqMan™ Advanced miRNA cDNA Synthesis Kit (Applied Biosystems, Cat no. A28007) following the manufacturer's instructions. PCR was carried out using TaqMan micro RNA advanced assay Pre-Amp master mix (Applied Biosystems, cat no.4391128 A) and TaqMan primers for -miR-16-5p (Assay ID 477860_mir), mir-146-5p (Assay ID 478399_mir), and hsa-miR-34c-5p (Assay ID 478052_mir). Expression of MiR-16-5p (assay ID 477860_mir) was used as a reference to normalize qPCR data. Relative expression of target miRNAs to the reference gene in each sample was calculated using an equation (2^A–B^/2^C–D^ [A = Ct value of the gene of interest at baseline in the first sample; B = Ct value of the gene of interest in each sample, C = Ct value of a reference gene in the first sample at baseline; and D = Ct value of a reference gene in each sample]). This gives the first control sample a relative expression of 1 and all other samples were calculated relative to this [51].

### 2.8 Statistics

Data are presented as mean ± SD (tables and text). Statistical analysis was performed with SPSS software (v24, IBM). Prior to primary analysis, normality of each variable was assessed with the Kolmogorov-Smirnov test and by visual analysis of histograms and plots for skew/kurtosis. Functional data are presented as individual data points at baseline vs. follow-up without statistical procedures (Suppl. Figs. S1-S3). Change following DQ in function, patient reported outcomes, and clinical and safety labs were evaluated with paired-samples *t*-test and baseline vs. follow-up bivariate Pearson correlation (single-arm design). The α-level for significance was 0.05. However, as this was a pilot investigation to determine outcomes of interest for a larger randomized placebo-controlled trial, trends are reported with an α-level of *p* ≤ .1.

Analyte data derived from microarray, multiplex, and ELISA were subjected to additional quality control and data transformations to reduce positive skew. Microarray data (MMPs, TIMPs, miRNAs) were log-transformed. For multiplexed cytokines and chemokines presented in Suppl. Table S4, samples with a proportion of null values (concentration below detection limits) exceeding 20% were omitted (FLT3L, MCP3, IL12p40, IL12p70, IL13, IL17a, IL7, TGFβ, VEGFα), and one statistical outlier removed (>80% multiplexed markers >3 SD). Cytokines, chemokines, and growth factors probed through exploratory multiplex were analyzed as inverse hyperbolic sine (asinh) transformed median fluorescence intensity luminex signals [52]. Plots of bivariate correlations of MFI signal against calculated concentrations were examined (raw and transformed). Change with DQ was evaluated by number of samples ≥5% improved for consistency with physical function, pulmonary function, and laboratory-based FI-LAB. Correlations are Pearson correlations (r) using transformed SASP markers (log or asinh). All correlations were confirmed visually by plots of baseline vs. follow-up. Associations with physical function, pulmonary function, and FI-LAB *r* ≥ 0.50 are shown in Table S4, and correspond to *p* ≤ .10.

## 3 Results

### 3.1 Patient recruitment, adherence, and completion of planned assessments

**Fig. 1:**
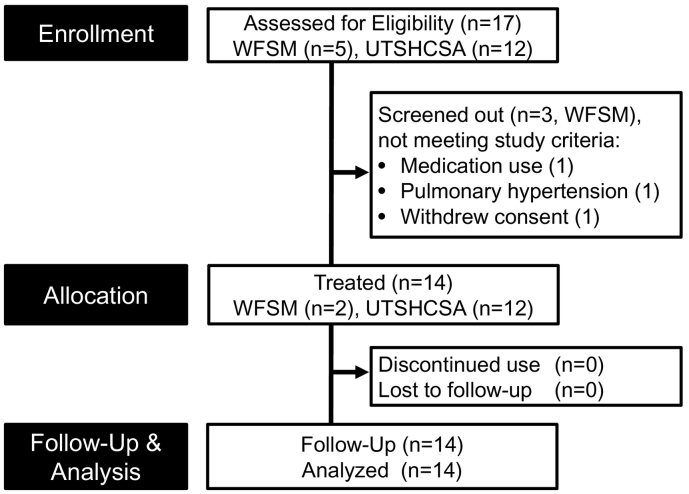
CONSORT flow diagram. Patient allocation in single-arm open label pilot study conducted at Wake Forest School of Medicine (WFSM) and University of Texas Health Science Center San Antonio (UTHSCSA) shown in CONSORT flowchart.

Fourteen participants (2 women) aged 70.8 ± 7.9 years with mild to severe but stable IPF (7 on nintedanib, 5 on pirfenidone, 2 not on antifibrotic therapy) were recruited (Table 1). The primary source of patient recruitment was through a study physician clinic. UTHSCSA was the primary recruitment site (*n* = 12). Of 17 participants screened in-clinic at both sites, 14 were eligible, underwent baseline assessments, and began the three-week long intermittent DQ drug course (Fig. 1). The majority of participants had moderate IPF (8 of 14 with FVC between 50 and 80% of predicted); 4 participants had mild disease (FVC >80% of predicted); and 2 participants had severe IPF (FVC <50% of predicted). The retention rate for completion was 100% (14 complete out of 14 enrolled), with no discontinuation of DQ. Adherence to the self-administered intermittent dosing schedule was 127/126 doses: 13 participants achieved perfect adherence (9/9 dosing days), whereas 1 participant unintentionally self-administered an additional dose (10/9 dosing days). Planned assessments were completed in 14/14 participants, except for an omitted SPPB at follow-up in 1 participant due to hospitalization for a likely respiratory infection.

**Table 1:** Patient characteristics.

N	14 (2 women)
Age, yrs. (mean, range)	70.8 (55–84)
MoCA, score (mean, range)	25.9 (22−30)

	*Number of patients*
Racial/ethnic category	
White	14
African American	0
Other	0
Latino or Hispanic	2
IPF severity by FVC	
Preserved (>90%)	1
Low (80–90%)	3
Moderate (50–80%)	8
Severe (<50%)	2
Medications	
*IPF-indicated medications*	
Nintedanib	7
Pirfenidone	5
None	2
*Non-IPF medications*⁎	
≤2	3
3-5	3
6–9	6
≥10	2
Comorbid conditions	
*Most frequent (≥5 patients*)	
Hypertension	8
Depression or Anxiety	7
Allergies	6
Hypothyroid	6
Insomnia or sleep disturbance	5
Diarrhea (Ofev-related)	6 (4)
*Number of conditions*	
2–3	5
4–6	5
≥7	4

⁎ Multivitamin/supplement and as needed OTC excluded.

**Fig. 2:**
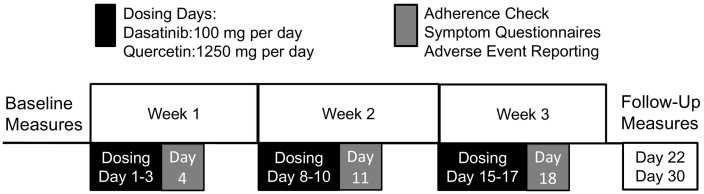
Three weeks of intermittent DQ dosing and assessment schedule. Dasatinib (100 mg per day) and Quercetin (1250 mg per day) were self-administered by participants for three consecutive days per week on three consecutive weeks according to the dosing schedule shown (dosing days 1–3, 8–10, and 15–17). Study coordinators called participant to confirm adherence and administer safety and symptom questionnaires approximately 24 h after the last dose of each week.

### 3.2 Safety and tolerability

Extensive symptom questionnaires were administered weekly. Symptom reports and diverse events that occurred during the study period are summarized in Table 2. In general, the majority of these events and symptoms were mild to moderate in severity, reversible, and without clinically significant sequelae, according to MedDRA v16.1. The most frequent events were respiratory symptoms (*n* = 14 reports; cough, shortness of breath, rhinorrhea), skin irritation and bruising from study procedures (n = 14 reports; 11 related to biopsy or adhesives), or gastrointestinal discomfort or heartburn (*n* = 12 reports). Two headache events resulted in temporary discontinuation of planned activities and were conservatively graded as “severe” according to pre-specified criteria. One serious adverse event was reported following completion of DQ intervention (possible bacterial multifocal pneumonia and pulmonary edema superimposed on IPF), which resulted in temporary hospitalization with subsequent complete resolution.

**Table 2:** Incidence and severity of adverse events.

Adverse events	Overall	Severity grade		
		Mild	Moderate	Severe
Non-serious Adverse Events	67	37	31	0
Serious Adverse Event	1	–	–	1
Fatal Adverse Event	0		–	–
Adverse Event leading to discontinuation	0		–	–

Reported adverse events				
IPF & respiratory (16 events)				
Cough	6	0	5	1*
Shortness of breath	3	0	2	1*
Runny nose, sneezing	**3**	3	0	0
Respiratory infection	1	0	0	1*
Low O_2_	1	0	1	0
Other respiratory (e.g. allergies)	2	2	0	0

General health & well-being (14 events)				
Feeling generally unwell	4	1	2	1*
Tired/weak	4	1	3	0
Nonspecific dizziness, light-headedness	3	3	0	0
Anxiety	2	0	2	0
Sleeplessness	1	1	0	0

Skin & bruising (14 events)				
Skin irritation (steri-strip)	10	9	1	0
Skin irritation (unrelated to steri-strip)	2	1	1	0
Bruising (biopsy site)	1	1	0	0
Bruising (unrelated to study procedure)	1	0	1	0

Gastrointestinal & appetite (14 events**)**				
Nausea	6	3	3	0
Change in appetite	2	2	0	0
Constipation	2	1	1	0
Diarrhea	2	1	1	0
Indigestion/heartburn	1	1	0	0
Vomiting	1	0	1	0

Pain or discomfort (7 events)				
Headaches	5	2	1	2
Joint pain or body discomfort	2	2	1	0

Mouth, eyes (5 events)				
Dry or watery eyes	3	2	1	0
Sore/sensitive mouth	2	1	2	0

Reported adverse events in the present trial were generally acceptable and largely consistent with events previously reported by participants in the placebo arms of randomized controlled trials in IPF [45,46]. Safety and tolerability with DQ are substantiated by acceptable safety profile of 9 months of daily D use in participants with systemic sclerosis with associated interstitial lung disease (SSc-ILD): mild-moderate severity diarrhea and nausea were most common reported adverse events [53]. However, similarly to the present pilot study, serious events involving edema, pleural effusion, and dyspnea were noted and possibly related to D superimposed on underlying SSc-ILD, though difficult to discern in a single-arm trial^53^. Future trials of DQ in IPF should carefully monitor such conditions.

Following three weeks of intermittent DQ administration, no differences were observed in body weight, vitals, or clinical chemistries (Suppl. Table S1).

### 3.3 Functional and biological measures

The most consistent improvements following DQ were observed in physical function (Table 3, Suppl. Fig. S3). Specifically, 6MWD, 4-m gait speed, chair-stands, and SPPB score were significantly improved when assessed one week after the completion of the drug administration (*p* < .05 all). The majority of participants exhibited physical function gains ≥5%. The 21.5-m increase in 6MWD is consistent with a clinically important improvement in IPF [54,55]. Of note, the 12 participants enrolled at UTHSCSA completed 6MWD tests in the clinic as part of their regular clinical assessments, which minimizes concerns that the improvement was attributable to a learning effect (Suppl. Methods). Changes with DQ were not observed in grip strength or spirometric measures (*r* > 0.90, Table 3, Suppl. Fig. S3). Eight of 14 participant's FI-LAB scores improved by at least 5%, but mean difference did not reach significance (*p* = .24, Table 3, Suppl. Fig. S1). When analyses were restricted to include only disease severity categorized as low to moderate (percent predicted FVC 5—90%, *n* = 11), results in this more homogenous group were similar to those of all subjects in our study (Suppl Table S2). Disease-specific health related quality of life and reported fatigue were unchanged (Table 4; p >0 .05 all).

**Table 3:** Changes in measures of physical and pulmonary function and frailty index derived from clinical chemistries before and after three-week intermittent DQ in 14 participants with stable IPF.

Functional measure	Baseline	Follow-up	Difference	Within-subjects	Correlation
	Mean ± SD	Mean ± SD	Δ ± SD	p-Value	r
Pulmonary function					
FEV1 (L/s)	2.1 ± 0.9	2.2 ± 0.7	+0.1 ± 0.3	0.38	0.95⁎
FEV1 (% predicted)	72.3 ± 26.3	73.4 ± 20.1	+1.14 ± 11	0.71	0.92⁎
FVC (L)	2.7 ± 1.0	2.6 ± 1.0	−0.2 ± 0.9	0.53	0.74⁎
FVC (% predicted)	67.3 ± 23.5	67 ± 17.9	−0.29 ± 9.1	0.91	0.94⁎
FEV1: FVC (%)	92.4 ± 8.3	91.6 ± 4.1	−0.7 ± 10	0.78	−0.27

Physical function					
6-min walk distance (m)	447 ± 83	468 ± 81	+21.5 ± 28	0.012⁎	0.94⁎
4-m gait speed (m/s)	1.1 ± 0.2	1.2 ± 0.2	+0.12 ± 0.2	0.024⁎	0.54⁎
Timed chair-stands (s)	14.8 ± 3	12.6 ± 2	−2.2 ± 3	0.013⁎	0.51⁎
SPPB score	10 ± 1	11 ± 0.9	+0.9 ± 1	0.003⁎	0.38
Grip strength (kg)	12.7 ± 5	12.1 ± 4	−0.6 ± 2	0.314	0.94⁎

Frailty index					
FI-LAB (score)⁎	0.103 ± 0.06	0.09 ± 0.07	0.02 ± 0.05	0.24	0.78⁎

⁎ *p* ≤ .05, *n* = 14 pulmonary function and 6-min walk test; *n* = 13 4-m gait speed, timed repeat chair-stands, SPPB score (1 subject did not complete due to coinciding health event), FEV1: forced expiratory volume in one *sec*ond (liters per sec, L/s); FVC: forced vital capacity (liters, L); Percent predicted values based on age, sex, race, and height. FI-LAB (lab-based frailty index score derived from analytes in/out of reference range for 34 blood-based clinical chemistries). Within-subjects, paired *t*-test (*p*-value) and Pearson correlations for baseline vs. follow-up.

**Table 4:** Changes in subjective respiratory health related quality of life and perceived fatigue before and after 3-week DQ.

Questionnaire	Baseline	Follow-up	Difference		Within-subjects	Corr
	Mean ± SD	Mean ± SD	Δ ± SD	No. ≥5% Improve	p-Value	r
Respiratory health						
MRC breathlessness	2.1 ± 0.8	2.3 ± 0.8	+0.2 ± 0.8	0		0.51
St. George's respiratory	26.9 ± 11	26.6 ± 12	−0.3 ± 5.4	5		0.89
UCSD shortness of breath	30.2 ± 17	28.6 ± 23	−3.6 ± 12	9		0.87

Perceived fatigue scales						
Pittsburgh fatigability, physical	24.4 ± 9	23.5 ± 8	−0.9 ± 5	4		0.80
Pittsburgh fatigability, mental	15.1 ± 15	12.1 ± 10	−3.0 ± 11	6		0.69
Fatigue severity	33.5 ± 13	21.1 ± 16	−2.3 ± 7	5		0.66
Fatigue visual analogue scale			−0.3 ± 3	5		

Global impression of change				**Improve**		
Overall change (Likert)	–	3.4 ± 2		6		
Global impression of change, VAS	–	4.4 ± 2		6		

*N* = 14. For each scale, a higher score indicates poorer perceived health or fatigue. Pearson correlations for baseline vs. follow-up. The Global Impression of Change administered at trial end to record change (if any) in activity limitations, symptoms, emotions, and overall quality of life related to IPF since study enrollment, queried by 2 inverse scales: a Likert scale (1 = no change, 7 = a great deal better), and Visual Analogue Scale (0 = much better, 10 = much worse). 6 patients indicated mild to moderate improvement; 7 patients report little/no noticeable change; 1 subject indicated “worsening” condition.

Circulating SASP factors were evaluated by multiplex, microarray, and targeted ELISA. Substantially larger sample sizes will be needed to definitively evaluate changes in biological markers of senescence and circulating SASP factors in patients with IPF since their circulating SASP factors assayed were not highly elevated initially (Suppl. Tables S3, S4). Though not statistically significant, select SASP proteins, including interluekin-6, matrix remodeling protein MMP7, and metallopeptidase inhibitor TIMP2, showed potential for reduction, with at least 8 participants having decreased circulating concentrations; however serum ELISA failed to confirm findings in IL-6 and MMP7. Moderate correlations were observed between change in physical function, pulmonary function, and FI-LAB and change in SASP factors (23 of 48 SASP factors, *r* ≥ 0.50; Suppl. Tables S3, S4). For example, gain in 6MWD was associated with lowered miR34c, improved 4 m-gait speed with MMP8, MMP9, and lowered RANTES, and faster chair-stands with decreased eotaxin, GRO-α, and interleukin-18. Though essentially unchanged with DQ, FEV1 or FVC were associated with lowered MCP-1 and with matrix remodeling proteins. Lowered FI-LAB was primarily associated with decreased pro-inflammatory cytokines. While provocative, these preliminary associations need to be confirmed in a larger population.

These functional and biological effects are generally consistent with preclinical findings in a murine bleomycin model of fibrotic lung disease, which supports our translational approach. Specifically, DQ initiated at disease onset in mice significantly reduced the senescence marker p16^INK4a^ expression in the lung and collective SASP factors (MCP1, IL6, TNFα, Col1a1, and TGFβ). Biological effects were matched by dramatic improvements in functional outcomes in lung-injured mice administered DQ, including a 54% relative improvement in median treadmill running endurance [5], which supports the clinical evidence reported here of meaningful change in physical function, even with modest changes in biomarkers of senescent cell burden.

## 4 Discussion

We report study feasibility in this first-in-human, single-arm, open-label pilot study of the senolytic drug combination, DQ, in participants with mild to severe but stable IPF. The three weeks of intermittent DQ administration was associated with clinically meaningful, statistically significant improvements in physical function. This included improved 6-min walk distance, 4-m gait speed, and 5-repeated chair-stand times. IPF appears to be relentlessly progressive: in large IPF drug trials, no improvements in 6MWD have been observed in the placebo-control arms [56]. These effects of DQ on physical function in IPF patients are consistent with preclinical findings of marked improvements in treadmill endurance and frailty following the selective ablation of senescent cells in progeroid, naturally aged, and lung-injured murine models [5,11,24,26]. Follow-up visits were conducted at least 5 days after the last dose of DQ, well beyond the elimination half-lives of D and Q, which are <6 h [43,44]. Thus, potential improvements in physical function did not depend on a mechanism that requires continued presence of the drugs (e.g., drug occupancy of a receptor or effects due to acting on an enzyme). Thus, potential improvements in physical function did not depend on a mechanism that requires continued presence of the drugs (e.g., drug occupancy of a receptor or effects due to acting on an enzyme). Rather, the improved physical function appears to depend on a mechanism that causes effects that persist following drug clearance (e.g., epigenetic effects or effects on tissue composition, such as a change in slowly turning-over cell types). However, the primary purpose of our study was not to evaluate drug efficacy: without a control group, the functional improvements we observed must be interpreted with caution and underscore the need for appropriately powered randomized controlled trials.

DQ administration was acceptable and feasible in the older IPF participants in this study. The single-arm open label study of three weeks of intermittent administration of DQ in older adults with stable IPF was successfully completed in all participants at two clinical sites. Drug adherence was imperfect but acceptable; routine dosing reminders and adherence checks by study personnel were seen as essential to ensure drug self-administration according to the intermittent dosing schedule. The senolytic agents were generally well tolerated without notable adverse changes in clinical chemistries. Specifically, there were no evident declines in renal or hepatic function or evidence of cell lysis syndrome. One hospitalization was reported following successful completion of DQ intervention, but the rest of the reported events were generally mild to moderate in severity, reversible, and without clinically significant sequelae. Though the single-arm open-label design precludes comparison of events against a placebo or standard of care control group as would be necessary to definitively evaluate safety and tolerability, we did compare event types and rate reporting to the placebo control arms of published Phase III randomized controlled trials in IPF (Suppl. Table S5). Overall, the majority of events are consistent with reports in these large drug trials in IPF [45,46], except for potentially higher reporting of cough, nausea, headache, and fatigue in this pilot trial. However, these speculations should be considered with caution, as differences among symptom questionnaires or severity potentially complicates direct comparison with these Phase III trials. We emphasize that physicians should not prescribe senolytics and IPF patients should not take these agents outside of clinical trials, unless and until further studies clearly demonstrate safety and efficacy.

Pulmonary function in this IPF patient population did not change during the course of this preliminary study. However, spirometry on a given day is a reflection of factors affecting lung function over the adult lifetime. For example, FEV1 and FVC can be improved acutely (e.g. with the use of bronchodilators), but for the most part these lung function parameters deteriorate with age and IPF disease course, and it takes the passage of time to characterize changing FVC trajectories. We did not hypothesize that the clearance of senescence cells would have an acute effect on bronchial reactivity, fibrosis, or extracellular matrix destruction. It is likely that in this pilot exploration, the follow-up period is too short and the sample size too modest to assess effects on long-term trajectories, especially in a complex chronic disease such as IPF. IPF etiology and progression is multifactorial, and whether the attendant pulmonary dysfunction is related to fibrosis vs. inflammation vs. other effects of senescent cells remains an open question [54]. For example, senescent cell-induced pulmonary dysfunction could be related to small vessel hemostasis due to serpines such as plasminogen activator inhibitor-1 (PAI-1), tissue destruction due to proteases, delayed tissue repair due to progenitor dysfunction caused by senescent cell production of activin A and other factors that cause stem/progenitor cell dysfunction, among other possibilities. Some of these potential mechanisms, each of which has been observed in other contexts, could take much longer to resolve following senescent cell reduction than others. If resolution of pulmonary scarring and fibrosis does indeed occur, it may take considerable time after clearance of senescent cells from the lung. In sum, a longer follow-up period in an efficacy trial that will enroll more participants and include a randomly-assigned control group will be needed to test if there is improvement in pulmonary function.

### 4.1 Limitations of the study

As clearly articulated throughout this report, a limitation in the single-arm open-label design is absence of a standard of care or placebo control arm. However, as this is a pilot study, the purpose was not to establish efficacy of DQ on functional outcomes, but to suggest feasibility and best methods to implement in a randomized, double-blind, controlled efficacy trial of senolytics for IPF or other senescence-associated age-related diseases. The improvements in physical function we found, although statistically significant and clinically meaningful, need to be verified in larger controlled trials. While efficacy and safety estimates should not be over-interpreted, our findings in older adults with stable IPF provide evidence supporting the conduct of such a trial of DQ with changes in physical function as a primary outcome.

Another limitation is in regards to markers of in vivo senescent cell burden for use in clinical trials. It is not feasible to test if senolytics directly cause senescent cell clearance from the lungs of IPF patients given safety concerns about repeated bronchoscopies. Our previous work demonstrated increased senescent cell abundance in lungs of subjects with IPF, including upregulation of p16^INK4A^ (CDKN2A), which correlated with disease severity [5]. Additionally, p16^INK4A+^ fibroblasts and epithelial cells with telomere-associated DNA damage foci (TAFs), another marker of cellular senescence, accumulated in fibrotic regions of honeycomb lung in patients with IPF, together with increased expression of SASP components (including the matrix remodeling proteins and pro-inflammatory factors that were tested in the blood of subjects in the present study) [5]. Moreover, using DQ we did demonstrate senescent cell clearance from lungs of mice with senescent cell accumulation and pulmonary fibrosis caused by inhaled bleomycin [5]. In exploratory analyses in the present pilot study, changes in circulating SASP factors following administration of DQ were inconclusive, possibly owing to the relatively low levels of these markers of cellular senescence at baseline in the circulation. However, we did find that circulating pro-inflammatory and pro-fibrotic SASP factors following 3 weeks of intermittent DQ administration were associated with change in physical function, pulmonary function, and FI-LAB scores. These findings need to be evaluated further in ongoing efforts to identify and validate reliable circulating biomarkers of cellular senescence that reflect tissue senescent cell abundance for use in multi-center clinical trials of senolytics, especially for diseases such as IPF in which repeated tissue biopsies are not feasible.

### 4.2 Drug and dosing considerations

Future trials should evaluate different senolytics and dosing regimens. We used intermittent DQ here because: 1) D and Q have been used extensively for other indications in humans and their safety profiles are understood, 2) DQ was effective in improving function in a mouse model of IPF [5], 3) DQ has been shown to be senolytic in human tissues [24], 4) DQ targets a broader range of senescent cells than some other senolytic regimens [8,42], and 5) DQ alleviates physical dysfunction, delays or treats multiple age-related disorders, and extends lifespan in old mice [5,8,11,18,[20], [21], [22], [23], [24], [25]]. We recently demonstrated that DQ causes selective apoptosis of senescent cells in freshly-isolated human tissue explants [24]. Alternative senolytic agents are being actively explored, including BCL-2 inhibitors and fisetin. We and others found that agents that target Bcl-xL, including navitoclax (ABT263), A1331852, and A1155463, are senolytic, at least with some types of senescent cells [18,57,58]. However, Bcl-xL inhibitors can interfere with neutrophil and platelet viability, sometimes causing severe thrombocytopenia even after small doses [59]. Fisetin is another agent that we discovered to be senolytic against some human cell types [18]. Like DQ, fisetin alleviates physical dysfunction in progeroid and naturally-aged mice, enhances healthspan, and increases remaining lifespan in older mice [26]. If fisetin proves to be safe and effective in mouse IPF models, it would be a strong candidate to advance into clinical trials, as may be other next-generation senolytics [8,42].

We used an intermittent dosing strategy for several reasons (see Methods). The development of the senescent phenotype is a relatively long and dynamic process that can take weeks, at least in cell culture [60]. However, senescent cell clearance by senolytics can occur within 18 h of a brief exposure, and senolytics do not need to be present continuously to occupy a receptor or affect an enzyme [5,8,42]. Rather, they act in a “hit-and-run” manner, which supports the rationale behind intermittent treatment [42]. To be conservative, we administered three courses of DQ separated by four days. Also, consistently with the above, we found improved physical function 5 days after the last dose of DQ, well after the day or so it takes for these agents to be completely eliminated. A substantial improvement in exercise endurance was also found in mice with pulmonary fibrosis intermittently treated with DQ [5], reflecting emerging evidence that brief or intermittent senolytic treatment results in profound effects that persist long after drug elimination.

### 4.3 Conclusions

IPF is a disease related to senescent cell accumulation in the lungs that is generally relentlessly progressive and fatal. While it remains to be confirmed if senolytics directly clear lung senescent cells to improve pulmonary function in humans with IPF as occurs in mice [5], our data in this first-in-human trial of senolytics indicate potential for clinically meaningful improvements in physical function. Consistent with the primary objective of this study, we established feasibility of recruitment, adherence, assessments, and methods. These findings constitute preliminary proof-of-concept evidence that interventions designed to target senescent cells may alleviate functional consequences of aging-related diseases in humans, as is the case in mice.

## Appendix A Supplementary data

Supplementary material 1 Image 1

Supplementary material 2 Image 2
