# Supplementary figures and images for: Senolytics in idiopathic pulmonary fibrosis: Results from a first-in-human, open-label, pilot study
Source: eBioMedicine. 2019 Jan 5;40:554–63. doi: 10.1016/j.ebiom.2018.12.052 (PMC6412088; doi:10.1016/j.ebiom.2018.12.052)

## Slide 1
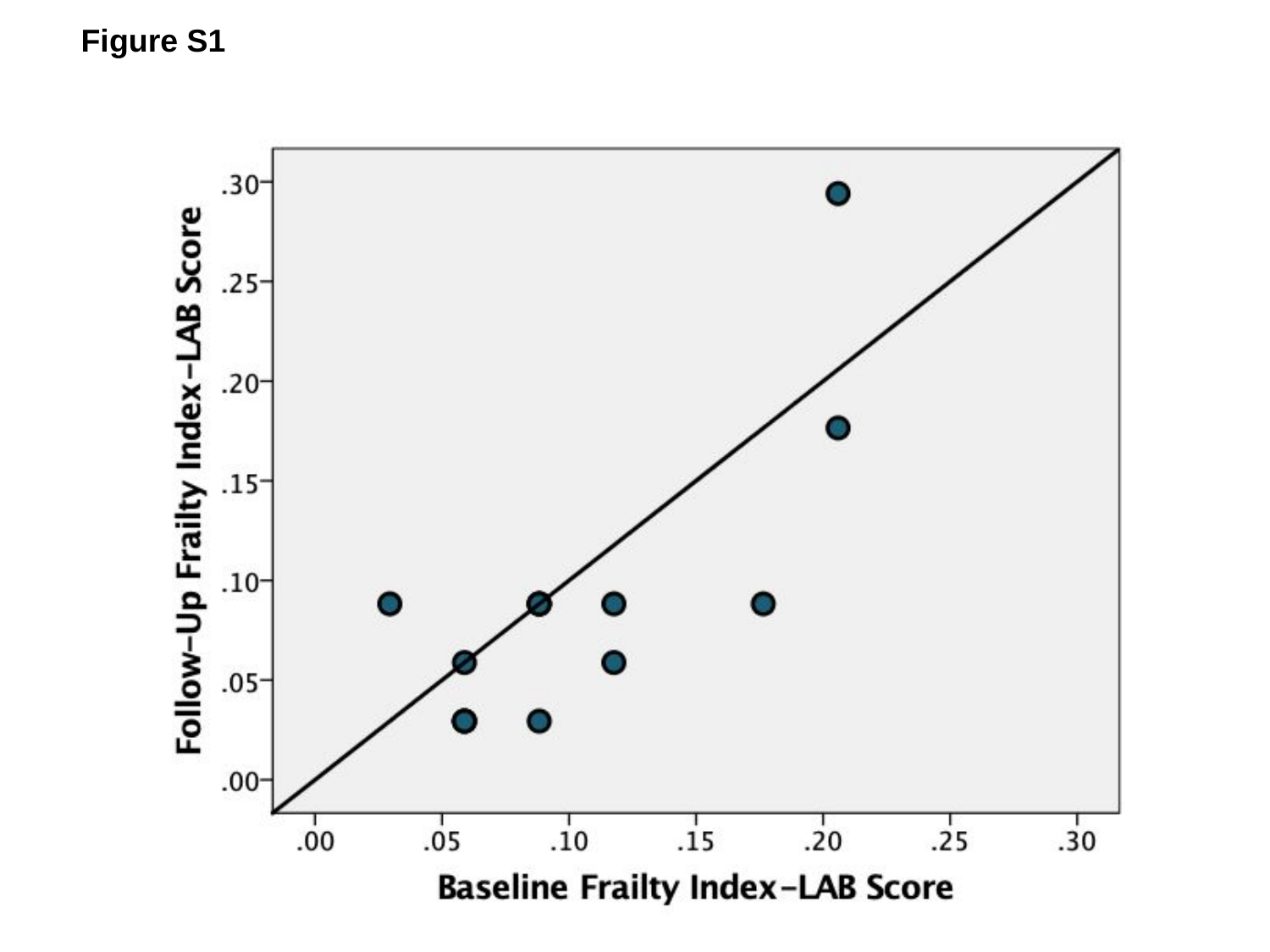

Figure S1

## Slide 2
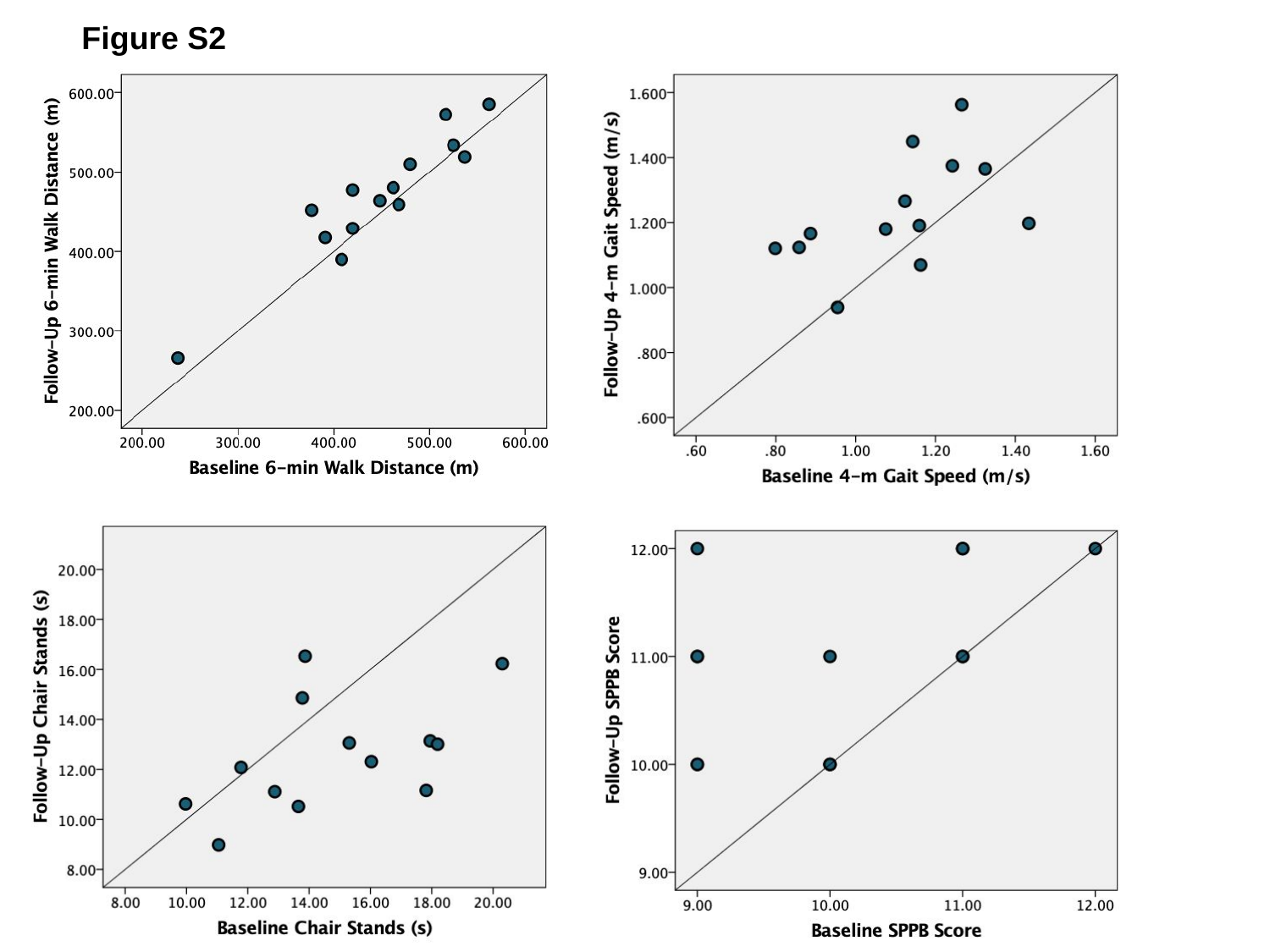

Figure S2

## Slide 3
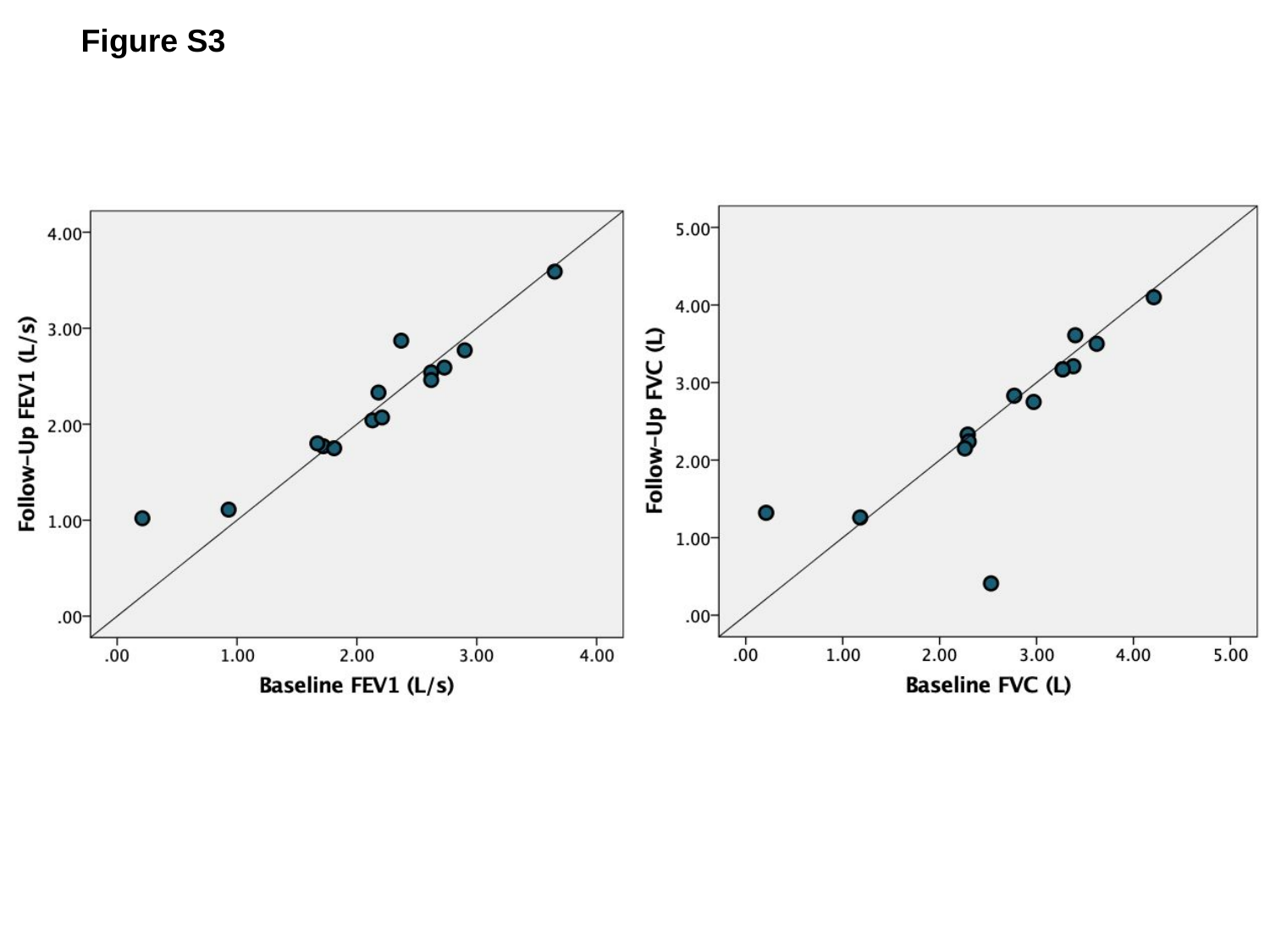

Figure S3

Supplement: Supplementary file 1 — Supplementary material 1 [file mmc1.pptx]
