# Supplementary material for: Senolytics in idiopathic pulmonary fibrosis: Results from a first-in-human, open-label, pilot study
Source: eBioMedicine. 2019 Jan 5;40:554–63. doi: 10.1016/j.ebiom.2018.12.052 (PMC6412088; doi:10.1016/j.ebiom.2018.12.052)
Supplement: Supplementary file 2 — Supplementary material 2 [file mmc2.docx]

**Supplemental Tables**

**Table S1.** Clinical and safety laboratory measures and serum inflammatory markers by ELISA before and after 3-weeks intermittent DQ administration. Pearson correlations (r) for baseline *vs*. follow-up.

| **Clinical & Safety** | **Baseline** | **Follow-Up** | **Difference** | **Within-Subject** | **Corr.** |
| --- | --- | --- | --- | --- | --- |
|  | **Mean ± SD** | **Mean ± SD** | **Δ ± SD** | **p-value** | **r** |
| Body weight (kg) | 88.3 ±14.2 | 88.0 ± 14 | -0.04 ± 0.6 | 0.786 | 0.99 |
| BMI | 28.9 ± 3.4 | 28.4 ± 3.5 | -0.02 ± 0.2 | 0.716 | 0.99 |
| SBP (mmHg) | 139 ± 13 | 144 ± 10 | 4.7±17 | 0.313 | 0.02 |
| DBP (mmHg) | 80.3 ± 10.7 | 80.8 ± 8.2 | 0.5 ± 7.8 | 0.814 | 0.69 |
| HbA1C (%) | 5.93 ± 0.9 | 5.98 ± 0.9 | 0.05 ± 0.13 | 0.187 | 0.99 |
| Glucose (mg/dL) | 106 ± 33 | 102 ± 23 | -3.6 ± 13.3 | 0.324 | 0.94 |
| **Lipids** |  |  |  |  |  |
| Total cholesterol (mg/dL) | 174 ± 30 | 168 ± 29 | -5.8 ± 26 | 0.428 | 0.61 |
| Triglycerides (mg/dL) | 162 ± 147 | 146 ± 122 | -16.8 ± 49 | 0.225 | 0.95 |
| HDL-C (mg/dL) | 49.6 ± 13.5 | 47.6 ± 13.2 | -1.9 ± 4.1 | 0.102 | 0.95 |
| LDL-C (mg/dL) | 95.0 ± 29.6 | 93.6 ± 36.8 | -1.3 ± 27.3 | 0.858 | 0.68 |
| **Kidney/ Liver** |  |  |  |  |  |
| Creatinine (mg/dL) | 0.96 ± 0.17 | 0.96 ±0.15 | 0.01 ± 0.1 | 0.936 | 0.82 |
| Bilirubin (mg/dL) | 0.66 ± 0.21 | 0.56 ± 0.22 | -0.10 ± 0.15 | 0.024 | 0.77 |
| Albumin (g/dL) | 4.18 ± 0.18 | 4.14 ± 0.26 | -0.04 ± 0.18 | 0.477 | 0.71 |
| ALT (IU/L) | 17.3 ± 8.0 | 19.6 ± 7.8 | 2.14 ± 4.9 | 0.127 | 0.81 |
| AST (IU/L) | 20.4 ± 5.1 | 20.9 ± 6.2 | 0.43 ± 2.7 | 0.56 | 0.91 |
| **Complete Blood Counts** |  |  |  |  |  |
| White blood cell count (x10e^3^/uL) | 7.44 ± 1.67 | 6.77 ± 1.74 | -0.66 ± 1.0 | 0.024 | 0.84 |
| Neutrophils (absolute, x10e^3^/uL) | 4.95 ± 1.56 | 4.32 ± 1.59 | -0.63 ± 0.92 | 0.024 | 0.83 |
| Lymphocytes (absolute, x10e^3^/uL) | 1.65 ± 0.61 | 1.64 ± 0.59 | -0.01 ± 0.29 | 0.854 | 0.89 |
| Monocytes (absolute, x10e^3^/uL) | 0.56 ± 1.45 | 0.64 ± 0.17 | 0.07 ± 0.9 | 0.012 | 0.85 |
| Red blood cell count (x10e^6^/uL) | 4.77 ± 0.59 | 4.60 ± 0.56 | -0.17 ± 0.3 | 0.041 | 0.88 |
| Hemoglobin (g/dL) | 15.2 ± 1.5 | 14.9 ± 1.2 | -0.3 ± 0.8 | 0.167 | 0.87 |
| Hematocrit (%) | 44.8 ± 4.1 | 43.7 ± 3.3 | -1.1 ± 2.4 | 0.124 | 0.82 |
| RBC Distribution Width (%) | 14.1 ± 0.9 | 14.1 ± 1.0 | 0.05 ± 0.4 | 0.655 | 0.91 |
| Mean Corpuscular Volume (fL) | 94.3 ± 5.8 | 94.9 ± 5.7 | 0.57 ± 1.4 | 0.149 | 0.97 |
| Mean Corpuscular Hemoglobin (pg) | 31.9 ±1.9 | 32.2 ± 2.0 | 0.35 ± 0.5 | 0.014 | 0.98 |
| **Inflammation Markers (ELISA)** |  |  |  |  |  |
| C-Reactive Protein (mg/L) | 1.96 ± 4.8 | 1.76 ± 3.8 | -0.21 ± 1.1 | 0.523 | 0.99 |
| Monocyte Chemoattr. Prot 1 (MCP1,ng/mL) | 212 ± 16 | 207 ± 14 | -5.9 ± 27 | 0.426 | 0.89 |
| Interleukin-6 (IL-6, pg/mL)* | 5.4 ± 1.4 | 4.4 ± 1.0 | -1.0 ± 3.7 | 0.312 | 0.73 |
| Plasminogen Actv. Inhib.1 (PAI-1, ng/mL)* | 3.10 ± 0.7 | 2.85 ± 0.6 | -0.25 ± 2.3 | 0.685 | 0.60 |
| Osteopontin (ng/mL) | 94.0 ± 4.8 | 96.2 ± 5.6 | 2.7 ± 17 | 0.568 | 0.70 |
| Apelin (ng/mL)* | 0.72 ± 0.05 | 0.68 ± 0.04 | -0.04 ± 0.2 | 0.401 | 0.52 |

*9 of 14 improve, score or level decrease ≥5%

**Table S2.** Changes in measures of physical and pulmonary function and frailty index derived from clinical chemistries before and after three-week intermittent DQ in 11 participants with stable IPF categorized as low-moderate severity (percent predicted FVC 50-90%).

| **Functional Measure** | **Baseline** | **Follow-Up** | **Difference** | **Within-**  **Subjects** | **Correlation** |
| --- | --- | --- | --- | --- | --- |
|  | **Mean ±SD** | **Mean ±SD** | **Δ ± SD** | **p-value** | **r** |
| **Pulmonary Function** |  |  |  |  |  |
| FEV1 (L/s) | 2.3 ± 0.57 | 2.3 ± 0.56 | +0.009 ± 0.19 | 0.88 | 0.94* |
| FVC (L) | 2.9 ±0.61 | 2.7 ± 0.97 | -0.24 ± 0.63 | 0.23 | 0.77* |
| **Physical Function** |  |  |  |  |  |
| 6-min walk distance (m) | 474 ± 57 | 498 ± 50 | +24.0 ± 29 | 0.019* | 0.87* |
| 4-m gait speed (m/s) | 1.14 ± 0.18 | 1.26 ± 0.16 | +0.12 ±0.17 | 0.046* | 0.48 |
| Timed chair-stands (s) | 14.0 ± 2.6 | 12.2 ± 2.1 | -1.8 ± 2.8 | 0.057^ | 0.31 |
| SPPB score | 10.3 ± 1.1 | 11.2 ± 0.9 | +0.91 ± 1.0 | 0.016* | 0.49* |
| **Frailty Index** | | | | | |
| FI-LAB (score)* | 0.83 ± 0.04 | 0.06 ± 0.03 | 0.02 ± 0.04 | 0.103 | 0.33 |

*p ≤ 0.05, ^p<0.10, n=11. FEV1: forced expiratory volume in one second (liters per sec, L/s); FVC: forced vital capacity (liters, L); FI-LAB (lab-based frailty index score derived from analytes in/out of reference range for 34 blood-based clinical chemistries). Within-subjects, paired t-test (p-value) and Pearson correlations for baseline *vs*. follow-up

**Table S3.** Log-transformed circulating matrix remodeling proteins and microRNA markers biological aging before and after 3-weeks intermittent DQ administration and associations with change in physical function, pulmonary function, and FI-LAB.

| **Marker** | **n** | **Baseline** | | **Follow-Up** | | **Within-Subjects** | **Pre-Post Correlation** | **No. ≥ 5%**  **Change w. DQ** | **Change with DQ: SASP vs. Function** |
| --- | --- | --- | --- | --- | --- | --- | --- | --- | --- |
|  |  | **Mean** | **SD** | **Mean** | **SD** | p-value | Pearson r |  | Pearson r |
| MMP1 | 11 | 2.63 | 0.32 | 2.75 | 0.27 | 0.086 | 0.90 | -- | FEV1, r = 0.52  FI-LAB Index, r= 0.59 |
| MMP2 | 12 | 4.62 | 0.16 | 4.62 | 0.14 | 0.939 | 0.84 | -- |  |
| MMP3 | 12 | 4.04 | 0.14 | 3.98 | 0.17 | 0.083 | 0.71 | 7 down | FEV1, r = 0.74 |
| MMP7 | 11 | 2.81 | 0.29 | 2.82 | 0.39 | 0.933 | 0.91 | 8 down |  |
| MMP8 | 11 | 2.72 | 0.33 | 2.46 | 0.43 | 0.142 | 0.54 | -- | 4m Gaitspeed, r = 0.72 |
| MMP9 | 12 | 4.78 | 0.23 | 4.73 | 0.26 | 0.443 | 0.76 | -- | 4m Gaitspeed, r = 0.61 |
| MMP10 | 11 | 3.04 | 0.55 | 3.05 | 0.48 | 0.967 | 0.16 | -- |  |
| MMP12 | 12 | 2.23 | 0.41 | 2.17 | 0.22 | 0.581 | 0.24 | 8 down |  |
| MMP13 | 12 | 3.32 | 0.24 | 3.14 | 0.15 | 0.04 | -0.13 | 8 down |  |
| TIMP1 | 12 | 4.76 | 0.06 | 4.75 | 0.06 | 0.892 | 0.79 | -- |  |
| TIMP2 | 12 | 4.92 | 0.08 | 4.93 | 0.08 | 0.052 | 0.96 | 8 up | FI-LAB Index, r = -0.72 |
| TIMP4 | 12 | 3.08 | 0.18 | 3.07 | 0.13 | 0.534 | 0.87 | -- | FEV1, r = 0.59 |
| mir146a | 14 | -0.26 | 0.67 | -0.11 | 0.42 | 0.503 | -0.18 | -- |  |
| mir126 | 14 | -0.13 | 0.36 | -0.01 | 0.15 | 0.241 | 0.37 | -- |  |
| mir34c | 10 | -0.77 | 0.90 | -0.47 | 0.66 | 0.137 | 0.93 | -- | 6MWD, r = -0.56 |

Change with DQ evaluated by plots of pre-post correlation and number ≥ 5% improved, *indicates paired samples t-test p ≤ 0.10.

Pre-Post Pearson r are correlations for log transformed microarray markers baseline *vs*. follow-up; all p<0.05.

Change with DQ: SASP *vs*. Function (right most column) shows Pearson correlations between percent change in log transformed marker with percent change in physical function (6MWD, 4m Gaitspeed, and 5-repeat chair-stand time), pulmonary function (FEV1, FVC), and Fi-LAB r ≥ 0.50, which correspond to p ≤ 0.10.

**Table S4.** Transformed (asinh) circulating multiplexed cytokines, chemokines, and growth factors with DQ and associations with change in physical function, pulmonary function, and FI-LAB.

| **Marker** | **Baseline** | | **Follow-Up** | | **Within-Subjects** | **Pre-Post Correlation** | **Change with DQ:**  **SASP vs. Function** |
| --- | --- | --- | --- | --- | --- | --- | --- |
| **LUMINEX** | **Mean** | **SD** | **Mean** | **SD** | **p-value** | **Pearson r** | **Pearson r** |
| EGF | 4.73 | 0.92 | 4.65 | 0.65 | 0.524 | 0.87 | FI-LAB, r = -0.54 |
| FGF2 | 4.37 | 0.33 | 4.34 | 0.26 | 0.624 | 0.85 | Chair-Stands, r = -0.73 |
| Eotaxin | 6.67 | 0.63 | 6.61 | 0.55 | 0.366 | 0.93 | Chair-Stands, r = -0.52 |
| TGFα | 4.12 | 0.31 | 4.10 | 0.34 | 0.616 | 0.91 |  |
| GCSF | 4.51 | 0.57 | 4.46 | 0.27 | 0.642 | 0.70 |  |
| GMCSF | 4.31 | 0.33 | 4.40 | 0.28 | 0.155 | 0.81 |  |
| Fractaline | 3.88 | 0.38 | 3.91 | 0.37 | 0.625 | 0.89 | FI-LAB, r = -0.75 |
| IFNA2 | 3.87 | 0.30 | 3.91 | 0.21 | 0.494 | 0.61 |  |
| INFgamma | 4.18 | 0.23 | 4.15 | 0.22 | 0.67 | 0.57 |  |
| GROalpha | 8.65 | 1.11 | 8.74 | 0.72 | 0.563 | 0.89 | Chair-Stands, r = -0.55 |
| IL10 | 4.37 | 0.45 | 4.41 | 0.31 | 0.732 | 0.60 | Chair-Stands, r= -0.57 |
| MCP3 | 5.72 | 0.84 | 5.72 | 0.79 | 0.469 | 0.98 |  |
| PDGFAA | 9.58 | 0.69 | 9.65 | 0.37 | 0.599 | 0.75 |  |
| PDGFBB | 8.66 | 0.81 | 8.77 | 0.50 | 0.481 | 0.90 |  |
| IL15 | 4.38 | 0.20 | 4.40 | 0.20 | 0.358 | 0.78 |  |
| sCD40L | 6.98 | 1.01 | 6.94 | 0.96 | 0.61 | 0.81 | 6MWD, r = -0.54  Chair-Stands, r =-0.53 |
| IL1RA | 4.70 | 0.56 | 4.69 | 0.54 | 0.827 | 0.95 |  |
| IL1a | 4.72 | 0.35 | 4.69 | 0.27 | 0.859 | 0.80 |  |
| IL9 | 4.11 | 0.31 | 4.15 | 0.27 | 0.592 | 0.67 | FI-LAB, r = -0.53 |
| IL1β | 4.15 | 0.29 | 4.13 | 0.29 | 0.518 | 0.84 | FI-LAB, r = -0.61 |
| IL2 | 4.31 | 0.24 | 4.33 | 0.21 | 0.566 | 0.73 | FI-LAB, r = -0.52 |
| IL3 | 3.97 | 0.26 | 3.98 | 0.24 | 0.622 | 0.67 | FI-LAB, r = -0.55 |
| IL4 | 4.12 | 0.37 | 4.12 | 0.32 | 0.931 | 0.69 | FI-LAB, r = -0.83 |
| IL5 | 4.48 | 0.61 | 4.53 | 0.56 | 0.961 | 0.96 |  |
| IL6 | 4.95 | 0.41 | 4.84 | 0.36 | 0.272 | 0.80 |  |
| IL8 | 5.93 | 0.54 | 5.88 | 0.47 | 0.13 | 0.90 |  |
| IP10 | 8.78 | 0.64 | 8.83 | 0.40 | 0.442 | 0.62 |  |
| MCP1 | 9.37 | 0.42 | 9.33 | 0.35 | 0.732 | 0.91 | FEV1, r = -0.65  FVC, r = -0.71 |
| MIP1a | 4.30 | 0.39 | 4.29 | 0.38 | 0.469 | 0.87 | FI-LAB, r = -0.55 |
| MIP1b | 4.54 | 0.25 | 4.55 | 0.24 | 0.828 | 0.82 |  |
| RANTES | 9.03 | 0.31 | 8.99 | 0.31 | 0.809 | 0.86 | 4m Gaitspeed, r = -0.55 |
| TNFα | 5.82 | 0.27 | 5.81 | 0.23 | 0.416 | 0.95 |  |
| IL18 | 5.78 | 0.51 | 5.78 | 0.36 | 0.196 | 0.87 | Chair-Stands, r = -0.72 |

Black: pro-inflammatory; Green: anti-inflammatory, Gray: undetermined.

Quality control: samples with a proportion of null values (concentration below detection limits) exceeding 20% are omitted (FLT3L, MCP3, IL12p40, IL12p70, IL13, IL17a, IL7, TGFβ, VEGFα). One statistical outlier removed (>80% markers >3 SD). Median fluorescence intensity signals asinh transformed. No marker levels changed with DQ |≥ 5%|. *indicates paired samples t-test p ≤ 0.10.

Pre-Post Pearson r are correlations for asinh baseline *vs*. follow-up; all p<0.05. Change with DQ: SASP *vs*. Function (right most column) shows Pearson correlations between percent change in asinh transformed multiplex analyte with percent change in physical function (6MWD, 4m Gait speed, and 5-repeat chair-stand time), pulmonary function (FEV1, FVC), and Fi-LAB r ≥ 0.50, which correspond to p ≤ 0.10.

| **Table S5.** Comparison of published most frequent adverse events reported in placebo arms of Phase III randomized control trials in IPF and events reported in the present single-arm, open-label pilot trial of senolytics in IPF. | | | | | | | | | | |
| --- | --- | --- | --- | --- | --- | --- | --- | --- | --- | --- |
|  | **Number of Reported Events** | | | | **Percent Patient Reporting Event** | | | | **No. Events** | **Percent Events** |
|  | **A+C** | **IN1** | **IN2** | **Combined** | **A+ C** | **IN1** | **IN2** | **Combined** | **IPF Pilot** | **IPF Pilot** |
| **N (total)** | **277** | **204** | **219** | **700** | **%** | **%** | **%** | **Avg %** | **14** | **%** |
| Cough | 82 | 26 | 31 | 139 | 29.6 | 12.7 | 14.2 | 18.8 | 6 | 42.9 |
| Nausea | 37 | 12 | 16 | 65 | 13.4 | 5.9 | 7.3 | 8.9 | 6 | 42.9 |
| Headache | 64 |  |  | 64 | 23.1 |  |  | 23.1 | 5 | 35.7 |
| Diarrhea | 60 | 38 | 40 | 138 | 21.7 | 18.6 | 18.3 | 19.5 | 2 | 14.3 |
| Upper respiratory tract infection | 56 | 18 | 24 | 98 | 20.2 | 8.8 | 11 | 13.3 | 1 | 7.1 |
| Fatigue | 48 |  |  | 48 | 17.3 |  |  | 17.3 | 4 | 28.6 |
| Rash | 24 |  |  | 24 | 8.7 |  |  | 8.7 | 0 | 0 |
| Dyspnea | 49 | 23 | 25 | 97 | 17.7 | 11.3 | 11.4 | 13.5 | 3 | 21.4 |
| Dizziness | 36 |  |  | 36 | 13 |  |  | 13 | 3 | 21.4 |
| IPF Exacerbation | 50 | 21 | 40 | 111 | 18.1 | 10.3 | 18.3 | 15.6 | 0 | 0 |
| Bronchitis | 36 | 28 | 17 | 81 | 13 | 13.7 | 7.8 | 11.5 | 0 | 0 |
| Constipation | 38 |  |  | 38 | 13.7 |  |  | 13.7 | 2 | 14.3 |
| Back pain | 37 |  |  | 37 | 13.4 |  |  | 13.4 | 2 | 14.3 |
| Dyspepsia^ | 17 |  |  | 17 | 6.1 |  |  | 6.1 | 1 | 7.1 |
| Nasopharyngitis | 30 | 34 | 34 | 98 | 10.8 | 16.7 | 15.5 | 14.3 | 3 | 21.4 |
| Anorexia | 18 | 14 | 10 | 42 | 6.5 | 6.9 | 4.6 | 6 | 2 | 14.3 |
| Vomiting | 24 | 4 | 7 | 35 | 8.7 | 2 | 3.2 | 4.6 | 1 | 7.1 |
| Decrease in weight | 22 | 13 | 2 | 37 | 7.9 | 6.4 | 0.9 | 5.1 | 0 | 0 |
| Gastroesophageal reflux | 18 |  |  |  | 6.5 |  |  | 6.5 | 1 | 7.1 |
| Insomnia | 18 |  |  |  | 6.5 |  |  | 6.5 | 1 | 7.1 |
| Severe adverse events |  | 37 | 62 | 99 |  | 18.1 | 28.3 | 23.2 | 2 | 14.29 |
| Serious adverse events |  | 55 | 72 | 127 |  | 27 | 32.9 | 30.0 | 1 | 7.14 |
| Fatal adverse events |  | 10 | 21 | 31 |  | 4.9 | 9.6 | 7.25 | 0 | 0 |
| Adverse events leading to treatment discontinuation |  | 22 | 33 | 55 |  | 10.8 | 15.1 | 13.0 | 0 | 0 |

A+C: Combined event data from placebo arms of ASCEND and CAPACITY Phase III Trials of Pirfenidone for IPF (aged 67.8±7.3 years, percent predicted FVC 68.6±10.9%). IN1 & IN2: Event data for INPULSIS1 (IN1) and INPULSIS2 (IN2) Phase III Trials of Nintedanib for IPF. IN1 participants were aged 66.9±8.2 years, and percent predicted FVC was 80.5±17.3% at baseline. IN2 patients aged 67.1±7.5 years, and percent predicted FVC was 78.1±19.0 % at baseline. Phase III trial events only reported as most frequent adverse events defined as those with an incidence of more than 10% in any study group; blank cells correspond to no published data due to infrequent event reporting.

IPF Pilot Events and Percent Events transferred from Table 2, collapsed by severity. Red shading indicates higher percent patients reporting event type in the present study compared with IPF patients on placebo in Phase III trials (difference >10%); yellow shading moderately elevated percent reporting compared with Phase III (difference within 5-10% higher), green shading similar (<5% difference) or better reporting in present trial compared with Phase III.

A+C reported in: King TE, Jr., Bradford WZ, Castro-Bernardini S, Fagan EA, Glaspole I, Glassberg MK, et al. A phase 3 trial of pirfenidone in patients with idiopathic pulmonary fibrosis. The New England journal of medicine. 2014 May 29;370(22):2083-92.

IN1 & IN2 reported in: Richeldi L, du Bois RM, Raghu G, Azuma A, Brown KK, Costabel U, et al. Efficacy and safety of nintedanib in idiopathic pulmonary fibrosis. The New England journal of medicine. 2014 May 29;370(22):2071-82.
